# All-optically phase-induced polarization modulation by means of holographic method

**DOI:** 10.1038/s41598-020-62549-z

**Published:** 2020-03-27

**Authors:** Ziyao Lyu, Changshun Wang

**Affiliations:** State Key Laboratory of Advanced Optical Communication Systems and Networks, School of Physics and Astronomy, Shanghai Jiao Tong University, Shanghai, 200240 China

**Keywords:** Nonlinear optics, Nonlinear optics

## Abstract

Phase-induced polarization modulation has been achieved experimentally by means of the all-optical holographic method. An extra spiral phase is added to a Gaussian beam and then a holographic grating is recorded through the interference of a Gaussian beam and the phase-vortex beam with the same linear polarization state in an azobenzene liquid-crystalline film. We report here that the polarization state of the diffraction light from the recorded grating is different from that of the incident light, while no polarization variation occurs for the holographic grating recorded by two Gaussian beams. The phase-induced polarization modulation is mainly attributed to the formation of birefringence in the film generated by phase vortex, which is investigated through the ripple patterns resulting from the competition between photoinduced torques and analysed by the Jones matrix. The experimental results could enrich the connotation between optical parameters and offer a method to realize polarization modulation through phase control.

## Introduction

In transverse waves, the polarization state characterizes how the electric field oscillates in the plane perpendicular to the propagation direction^1^. Because optical communications and light-matter interactions strongly depend on the polarization, it is always desirable to manipulate the polarization state flexibly in a wide range of fields including microwave communication systems, liquid crystal display and many optical instruments^2–4^. In recent years, an increasing number of researches have focused on this topic. For example, a polarization modulation scheme of electromagnetic waves was proposed through the reflection of a tunable metamaterial reflector and absorber^5^. The possibility of achieving laser emission with a desired polarization was also realized through the microfiber^6^. Moreover, a new polarization modulation scheme based on an inherently stable interferometer was reported as well^7^. On the other hand, phase is another essential parameter of light with many applications, e.g., the Zernike microscope^8^. In the last few decades, phase vortex, i.e., light carrying orbital angular momentum (OAM), has attracted great attention. Like polarization and wavelength, OAM provides an additional degree of freedom, which can be of great benefit in the fields of optical processing, communications and imaging systems^9–11^. Phase vortex is able to exist in a Gaussian beam which is given a number *m*, called the topological charge (TC)^12,13^. TC represents the number of 2π phase cycles when the optical phase circles once the beam axis. The transverse cross-sections of the optical vortices are associated with helical phase wavefront^14,15^. In many cases, there is no connection between the polarization and phase vortex of light.

In order to all-optically manipulate the polarization through the phase, the material with light-controlled properties is indispensable^16^. Azobenzene-containing polymers have become attractive because of the photoinduction of optical anisotropy and the generation of holographic gratings through the photoinduced reorientation^17–21^. During the holographic recording process, the azobenzene polymer is illuminated by two or more polarized interference beams, leading to the azobenzene groups reorienting perpendicularly to the polarization direction of light field and the formation of the photoinduced anisotropy, which is believed to result from the *trans–cis–trans* isomerization cycles of the azo-unit^22,23^. Particularly, azobenzene side-chain liquid-crystalline polymers have been found to be attractive owing to their large photoinduced birefringence and long-term optical storage^24,25^, so that more attention has been paid to the field of optical control with this kind of materials^26^.

In this work, phase-induced polarization modulation has been achieved through the holographic technique experimentally in azobenzene liquid crystals (ALC), which has not been studied adequately. Typically, the polarization state of the diffraction light is not able to be modulated in terms of the holographic grating recorded by the interference of two Gaussian beams with the same polarization state. In contrast, an extra spiral phase is added to one of the recording beams and a vortex-based grating (VBG) is generated within the interference light field carrying the phase vortex. It is found that the polarization state of the diffraction light from VBG changes asynchronously with the incident polarization state being manipulated, indicating that the optical polarization state is modulated by the recorded grating. The phase-induced polarization modulation is analyzed through Jones matrices and the mechanism is mainly attributed to the vortex-induced birefringence in the ALC film.

## Holographic Recording and Polarization Modulation Matrix

### Holographic recording

**Figure 1:**
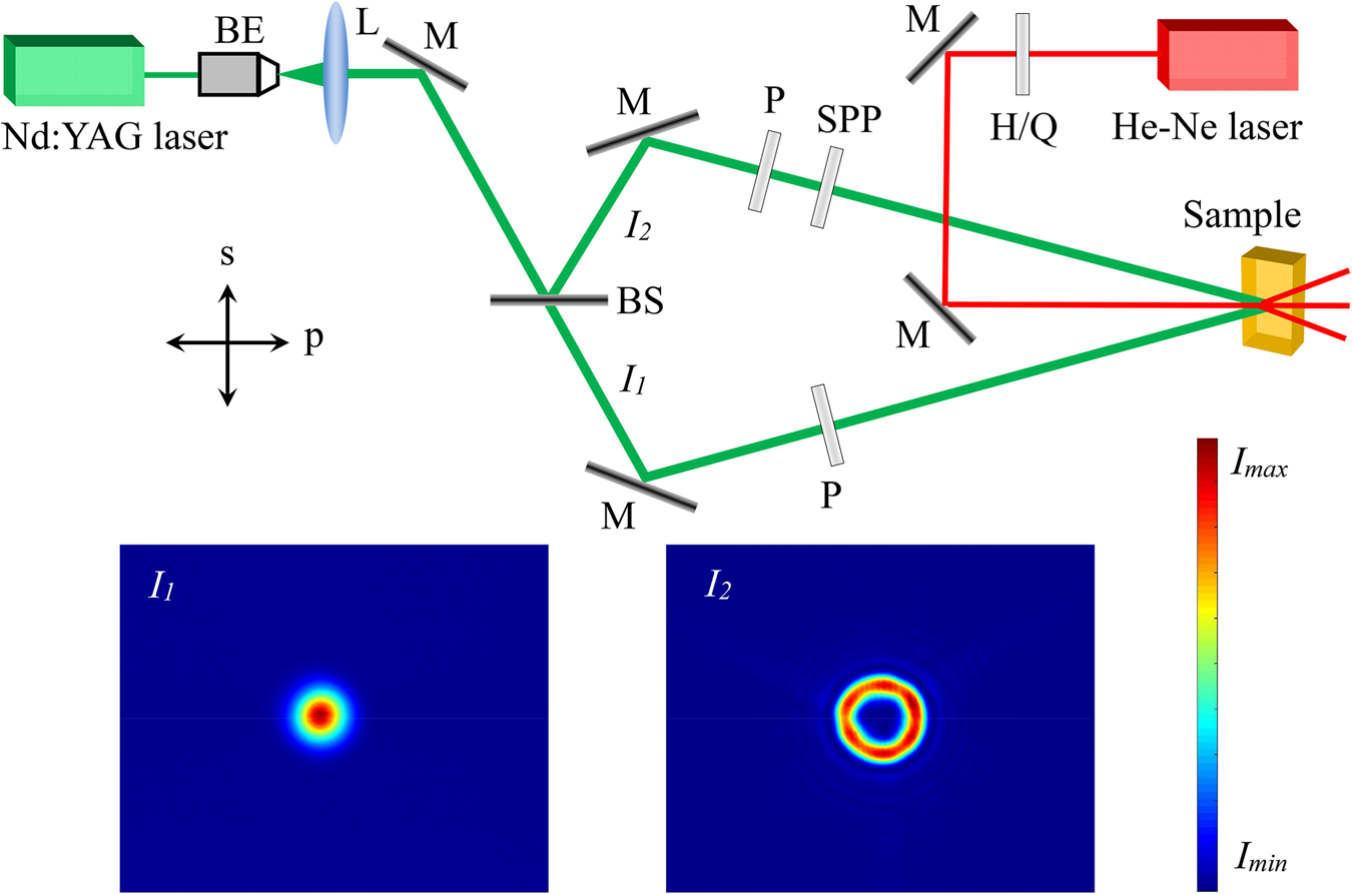
Pump-probe experimental setup of holographic recording for phase-induced polarization modulation. BE, beam expander; L, collimating lens; Q, quarter-wave plate; H, half-wave plate; P, linear polarizer; BS, beam splitter; SPP, spiral phase plate; M, mirror. s and p represent the vertical and horizontal directions, respectively. Inset: spatial intensity distributions of the two recording beams detected by CCD.

The experimental setup is schematically presented in Fig. 1 to realize phase-induced polarization modulation. A 532 nm beam with the power density of 250 mW/cm^2^ from a Nd:YAG laser is applied as the pump light. The beam passes through a beam expander and a collimating lens in order to form the plane wave (the diameter of the light spot behind the lens is about 4 mm)^27^. The pump light is divided into two recording beams, *I*_1_ and *I*_2_, through a beam splitter (the intensity ration between *I*_1_ and *I*_2_ is 1:3) and then an extra phase is added to *I*_2_ through a spiral phase plate (Well optics, SPP-532-3-S10) (TC = 3) without changing the polarization state. Two polarizers are employed to make both recording beams s-linearly polarized (s and p directions are presented in Fig. 1). The two recording beams interfere at the surface of the ALC film with an intersection angle of 20° and the distance between the spiral phase plate and the sample surface is about 1.2 m. The recording time is 30 s. The sample is a kind of supermolecular materials synthesized through the ionic self-assembly of poly ionic liquid and azobenzene dyes. The charged polymer poly (1-butyl-vinylpyridinium bromide) is selected as the main chain, and the methyl orange dye is selected as the building unit. The thickness of the ALC film is 20 μm. Then, the recorded VBG is investigated by a 633 nm Gaussian beam from a He-Ne laser with the power density of 150 mW/cm^2^. The diameter of the probe light is 4 mm. The probe light is s-linearly polarized initially and the polarization state can be controlled through the wave plates, which is measured with a free-space polarimeter (THORLABS, PAX5710VIS-T). All measurements are performed at room temperature.

### Polarization modulation matrix of VBG

The polarization modulation matrix of the recorded VBG is discussed. The Jones vectors of the two recording beams can be expressed as ${{\boldsymbol{E}}}_{{\bf{1}}}={(0{E}_{1})}^{T}\exp (i\delta /2)$ and ${{\boldsymbol{E}}}_{{\bf{2}}}={(0{E}_{2})}^{T}\exp (-i\delta /2)\exp (im\varphi )$, respectively, with *δ* being the phase difference between the two recording beams caused by the optical path difference, *imφ* being introduced by the extra spiral phase and (0 1)^*T*^ representing the s-linear polarization state. The interference field is the sum of *E*_1_ and *E*_2_

1 $${\boldsymbol{E}}=\exp \left(-\frac{i\delta }{2}\right)(\begin{array}{c}0\\ {E}_{{1}}\,\exp (i\delta )+{E}_{{2}}\,\exp (im\varphi )\end{array})$$

**Figure 2:**
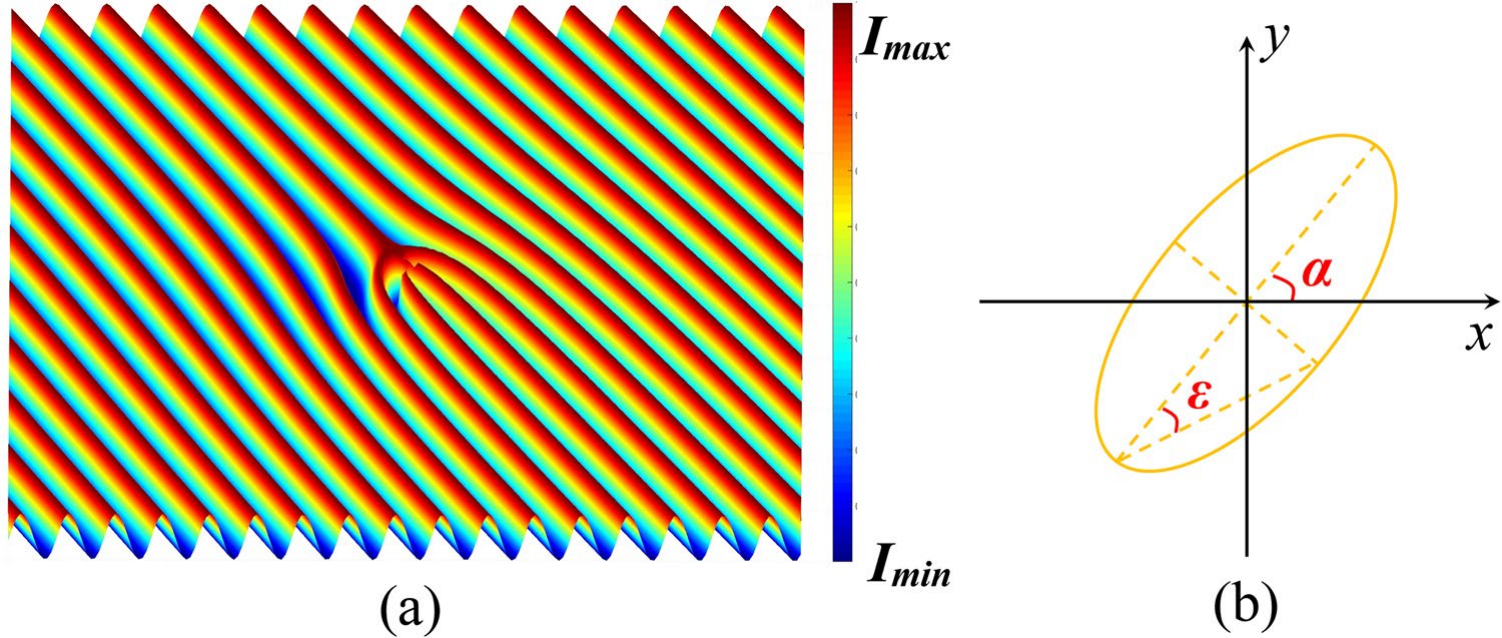
(**a**) Fork-shaped light intensity distribution of the vortex-Gaussian interference field. The interference pattern is stored in the ALC film, leading to the formation of VBG. (**b**) The polarization state of light can be illustrated by a polarization ellipse. *α* and *ε* are the azimuth and ellipticity, respectively.

The light intensity distribution describe by Eq. 1 is shown in Fig. 2(a). It is worth mentioning that the intensity distribution in the interference field is fork-shaped^28–30^, while the structure of VBG is not exactly consistent with the light distribution because of the competition between photoinduced torques discussed below. The photoinduced refractive index ***n***_***p***_ in the ALC film is $${{\boldsymbol{n}}}_{{\boldsymbol{p}}}=(\begin{array}{cc}{n}_{1} & 0\\ 0 & {n}_{2}\end{array})$$, where *n*_1_ and *n*_2_ are in the directions parallel and perpendicular to the major axis of the polarization ellipse, respectively. In the s-p coordinate system, the refractive index changes to ${{\boldsymbol{n}}}_{{\boldsymbol{p0}}}={\boldsymbol{R}}(\,-\,{\gamma })\cdot {{\boldsymbol{n}}}_{{\boldsymbol{p}}}\cdot {\boldsymbol{R}}({\gamma })$ with ***R*** being the rotation matrix and *γ* being the intersection angle between the major axis of the polarization ellipse and p-direction. The transmission function of VBG is ***t*** = exp[*i*2π*d*(***n***_***i***_ + ***n***_***po***_)/*λ*] with ***n***_***i***_ and *d* being the initial refractive index and thickness of the sample, respectively, and *λ* being the wavelength of the incident light^31^. The polarization modulation matrix of the VBG has the form

2 $${{\boldsymbol{T}}}_{{\boldsymbol{VBG}}}=(\begin{array}{cc}{g}_{1} & {g}_{2}\\ {g}_{2} & {g}_{3}\end{array})$$

For an arbitrarily polarized incident beam, the Jones vector of the polarization state is

3 $${{\boldsymbol{P}}}_{{\boldsymbol{in}}}=(\begin{array}{c}\cos \,\alpha \,\cos \,\varepsilon -i\,\sin \,\alpha \,\sin \,\varepsilon \\ \sin \,\alpha \,\cos \,\varepsilon +i\,\cos \,\alpha \,\sin \,\varepsilon \end{array})$$

where *α* and *ε* are the azimuth and ellipticity, respectively, as shown in Fig. 2(b) ^32^. When the polarization state of the diffraction light ***P***_***out***_ is detected, ***T***_***VBG***_ can be expressed as

4 $${{\boldsymbol{T}}}_{{\boldsymbol{VBG}}}=\frac{{{\boldsymbol{P}}}_{{\boldsymbol{out}}}\cdot {{\boldsymbol{P}}}_{{\boldsymbol{in}}}^{\ast }}{{|{{\boldsymbol{P}}}_{{\boldsymbol{in}}}|}^{2}}$$

*g*_*1*_, *g*_*2*_ and *g*_3_ will be determined through Eq. 4 with the experimental data in the next section.

## Results and discussion

### Polarization modulation under the condition of linearly polarized incident light

**Figure 3:**
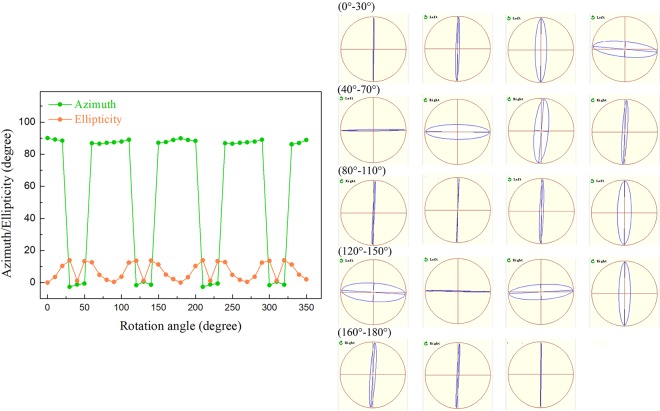
Polarization variation behavior of the diffraction beam as the optical axis of the half-wave plate is rotated from 0° to 350°. The arrow at the upper left corner of each polarization state represents the polarization direction. “Left” and “Right” are the left- and right-handed polarization states, respectively. The arrow is not displayed when the light is linearly polarized.

Here, we rotate the half-wave plate in a circle at an interval of 10° (the rotation angle is *β*) to manipulate the azimuth of the linearly polarized probe light. We set *ψ* being the angle between the fast axis of the wave plate and p-direction^33^, and the fast axis of the half-wave plate is in the s-direction (*ψ* = 90°) initially. The modulated polarization states of the diffraction light from VBG are summarized in Fig. 3. With the half-wave plate being rotated a circle, the diffraction light is not always linearly polarized and the range of |*ε* | detected by the free-space polarimeter is between 0° and 16.6°. When the light is right-handed elliptically polarized, *ε* > 0. On the other hand, *ε* < 0 under the condition of left-handed elliptical polarization states. In terms of the azimuth, *α* mainly concentrates in two regions, 87.8° ± 2.2° and 0° ± 1.6°. According to Fig. 3, there are four peaks along the ellipticity curve that all located exactly at the rapid changing parts of the azimuth curve in one period (0° < *β* < 180°), indicating that the azimuth conversion (0°→90°→0°) corresponds to the linear-elliptical-linear polarization variation. Moreover, the polarization direction of the diffraction light also varies periodically. The diffraction light is left-handed polarized first and changes to right-handed polarized at *β* = 40°. When *β* = 90°, the polarization state is back to be left-handed and becomes right-handed again at *β* = 130°. Accordingly, the peaks of the ellipticity curve represent the transformation of the azimuth and the valleys correspond to the change of the polarization direction. The polarization modulation matrix of VBG can also be obtained with the experimental data. First, we consider the condition that the diffraction light is right-handed elliptically polarized (*ε* > 0). The equation of polarization modulation is ${{\boldsymbol{P}}}_{{\boldsymbol{out}}}={{\boldsymbol{T}}}_{{\boldsymbol{VBG}}}\cdot {{\boldsymbol{T}}}_{1{\boldsymbol{/}}2}\cdot {{\boldsymbol{P}}}_{{\boldsymbol{probe}}}$

5 $$(\begin{array}{c}\cos \,\alpha \,\cos |\varepsilon |-i\,\sin \,\alpha \,\sin |\varepsilon |\\ \sin \,\alpha \,\cos |\varepsilon |+i\,\cos \,\alpha \,\sin |\varepsilon |\end{array})=(\begin{array}{cc}{g}_{1} & {g}_{2}\\ {g}_{2} & {g}_{3}\end{array})\cdot (\begin{array}{cc}\cos \,2\psi  & \sin \,2\psi \\ \sin \,2\psi  & -\,\cos \,2\psi \end{array})\cdot (\begin{array}{c}0\\ 1\end{array})$$

6 $${{\boldsymbol{T}}}_{{\boldsymbol{VBG}}}=\left(\begin{array}{cc}\frac{{g}_{2}\,\cos \,2\psi +\,\cos \,\alpha \,\cos |\varepsilon |-i\,\sin \,\alpha \,\sin |\varepsilon |}{\sin \,2\psi } & {g}_{2}\\ {g}_{2} & \frac{{g}_{2}\,\sin \,2\psi -\,\sin \,\alpha \,\cos |\varepsilon |-i\,\cos \,\alpha \,\sin |\varepsilon |}{\cos \,2\psi }\end{array}\right)$$

where ***T***_***1/*2**_ is the transmission matrix of the half-wave plate^33^. From Eq. 6, only one element *g*_2_ exists in ***T***_***VBG***_ and *g*_2_ can be determined through matrix normalization. Similarly, when the diffraction light is left-handed elliptically polarized (*ε* < 0), the polarization modulation matrix changes to

7 $${{\boldsymbol{T}}}_{{\boldsymbol{VBG}}}=\left(\begin{array}{cc}\frac{{g}_{2}\,\cos \,2\psi +\,\cos \,\alpha \,\cos |\varepsilon |+i\,\sin \,\alpha \,\sin |\varepsilon |}{\sin \,2\psi } & {g}_{2}\\ {g}_{2} & \frac{{g}_{2}\,\sin \,2\psi -\,\sin \,\alpha \,\cos |\varepsilon |+i\,\cos \,\alpha \,\sin |\varepsilon |}{\cos \,2\psi }\end{array}\right)$$

The values of *g*_2_ in different linear polarization situations are listed in Table 1. According to Table 1, *g*_2_ changes with the polarization state of the incident light. The reason is that VBG causes the depolarization of the incident light and the degree of polarization (DoP) of the diffraction light measured by the free-space polarimeter changes under different incident polarization conditions. According to Eq. (4), the variation of DoP is reflected by ***T***_***VBG***_ and *g*_2_ is not constant as the incident polarization state is modulated.

**Table 1:** Dependence of *g*_2_ and  DoP on the rotation angle *β* of the half-wave plate.

*β* = 0°	DoP = 98.1%	*g*_2_ = 0	*β* = 90°	DoP=98.3%	*g*_2_ = 0
*β* = 10°	DoP = 82.7%	*g*_2_ = 0.34 + 0.07*i*	*β* = 100°	DoP=83.2%	*g*_2_ = 0.34 + 0.08*i*
*β* = 20°	DoP = 73.5%	*g*_2_ = 0.68 + 0.13*i*	*β* = 110°	DoP=71.2%	*g*_2_ = 0.67 + 0.14*i*
*β* = 30°	DoP = 67.9%	*g*_2_ = −0.46 + 0.34*i*	*β* = 120°	DoP=65.8%	*g*_2_ = −0.47 + 0.34*i*
*β* = 40°	DoP = 92.8%	*g*_2_ = −0.16 + 0.05*i*	*β* = 130°	DoP=93.1%	*g*_2_ = −0.17 + 0.04*i*
*β* = 50°	DoP = 63.2%	*g*_2_ = 0.17–0.25*i*	*β* = 140°	DoP=61.9%	*g*_2_ = 0.18–0.24*i*
*β* = 60°	DoP = 71.9%	*g*_2_ = 0.63 + 0.69*i*	*β* = 150°	DoP=70.3%	*g*_2_ = 0.61 + 0.68*i*
*β* = 70°	DoP = 82.0%	*g*_2_ = 0.62 + 0.16*i*	*β* = 160°	DoP=82.6%	*g*_2_ = 0.62 + 0.16*i*
*β* = 80°	DoP = 85.3%	*g*_2_ = 0.39 + 0.04*i*	*β* = 170°	DoP=85.5%	*g*_2_ = 0.38 + 0.04*i*

### Polarization modulation under the condition of elliptically polarized incident light

**Figure 4:**
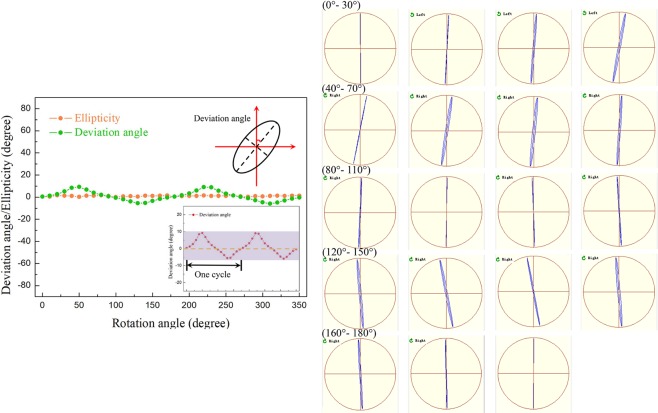
Dependence of the deviation angle and ellipticity of the modulated polarization state on the rotation angle of the quarter-wave plate from 0° to 350°. Inset: the diagram of the deviation angle and the curve of the deviation angle in the range of ±20°.

Then, we employ a single quarter-wave plate to control the ellipticity of the polarized probe light. The range and interval of the rotation angle are selected the same as the experiment above. Images and summarization of the phase-modulated polarization states with the fast axis of the quarter-wave plate being modulated from 0° to 350° are presented in Fig. 4. Given the major axis of the polarization ellipse swinging around *y*-axis, another parameter, deviation angle (the intersection angle between the major axis of polarization ellipse and *y*-axis), is introduced and illustrated in the inset of Fig. 4. As the quarter-wave plate is rotated, the deviation angle changes from 0° to 8.7°, moves backwards to −5.9° and returns to 0° in one period. From the inset of Fig. 4, it can be noticed that the major axis of the polarization ellipse moves more rapidly when the deviation angle becomes larger. Moreover, the diffraction light is nearly linearly polarized with |*ε* | keeping less than 1.8° during the whole process. Similarly, the polarization modulation matrix can be obtained through ${{\boldsymbol{P}}}_{{\boldsymbol{out}}}={{\boldsymbol{T}}}_{{\boldsymbol{VBG}}}\cdot {{\boldsymbol{T}}}_{1{\boldsymbol{/}}4}\cdot {{\boldsymbol{P}}}_{{\boldsymbol{probe}}}$

8 $$(\begin{array}{c}\cos \,\alpha \,\cos \,\varepsilon -i\,\sin \,\alpha \,\sin \,\varepsilon \\ \sin \,\alpha \,\cos \,\varepsilon +i\,\cos \,\alpha \,\sin \,\varepsilon \end{array})=(\begin{array}{cc}{g}_{1} & {g}_{2}\\ {g}_{2} & {g}_{3}\end{array})\cdot (\begin{array}{cc}{\cos }^{2}\psi +i{\sin }^{2}\psi  & (1-i)\sin \,\psi \,\cos \,\psi \\ (1-i)\sin \,\psi \,\cos \,\psi  & {\sin }^{2}\psi +i{\cos }^{2}\psi \end{array})\cdot (\begin{array}{c}0\\ 1\end{array})$$

The values of *g*_2_ and DoP in different ellipticity situations are shown in Table 2. Similarly, *g*_2_ varies with the change of DoP as the incident polarization state is modulated by the quarter-wave plate.

**Table 2:** Dependence of *g*_2_ and DoP on the rotation angle *β* of the quarter-wave plate.

*β* = 0°	DoP = 98.2%	*g*_2_ = 0	*β* = 90°	DoP=98.8%	*g*_2_ = 0
*β* = 10°	DoP = 89.1%	*g*_2_ = 0.19–0.17*i*	*β* = 100°	DoP=90.2%	*g*_2_ = 0.17–0.18*i*
*β* = 20°	DoP = 81.2%	*g*_2_ = 0.37–0.33*i*	*β* = 110°	DoP=83.5%	*g*_2_ = 0.32–0.36*i*
*β* = 30°	DoP = 72.7%	*g*_2_ = 0.54–0.47*i*	*β* = 120°	DoP=73.1%	*g*_2_ = 0.46–0.51*i*
*β* = 40°	DoP = 65.8%	*g*_2_ = 0.67–0.62*i*	*β* = 130°	DoP=67.3%	*g*_2_ = 0.57–0.61*i*
*β* = 50°	DoP = 64.3%	*g*_2_ = 0.63–0.68*i*	*β* = 140°	DoP=65.9%	*g*_2_ = 0.60–0.57*i*
*β* = 60°	DoP = 74.8%	*g*_2_ = 0.48–0.58*i*	*β* = 150°	DoP=75.2%	*g*_2_ = 0.51–0.46*i*
*β* = 70°	DoP = 80.3%	*g*_2_ = 0.33–0.40*i*	*β* = 160°	DoP=82.6%	*g*_2_ = 0.35–0.33*i*
*β* = 80°	DoP = 87.4%	*g*_2_ = 0.17–0.21*i*	*β* = 170°	DoP=89.1%	*g*_2_ = 0.18–0.17*i*

Based on the values of *g*_*2*_ in Tables 1 and 2, the variation curves of *g*_1_, *g*_2_ and *g*_3_ can be fitted and ***T***_***VBG***_ is obtained. According to the experimental results above, as an extra spiral phase is added to the recording field, the polarization state of the diffraction light from VBG is able to be modulated except for the condition that the probe light is s-linearly polarized.

### Mechanism of phase-induced polarization modulation

**Figure 5:**
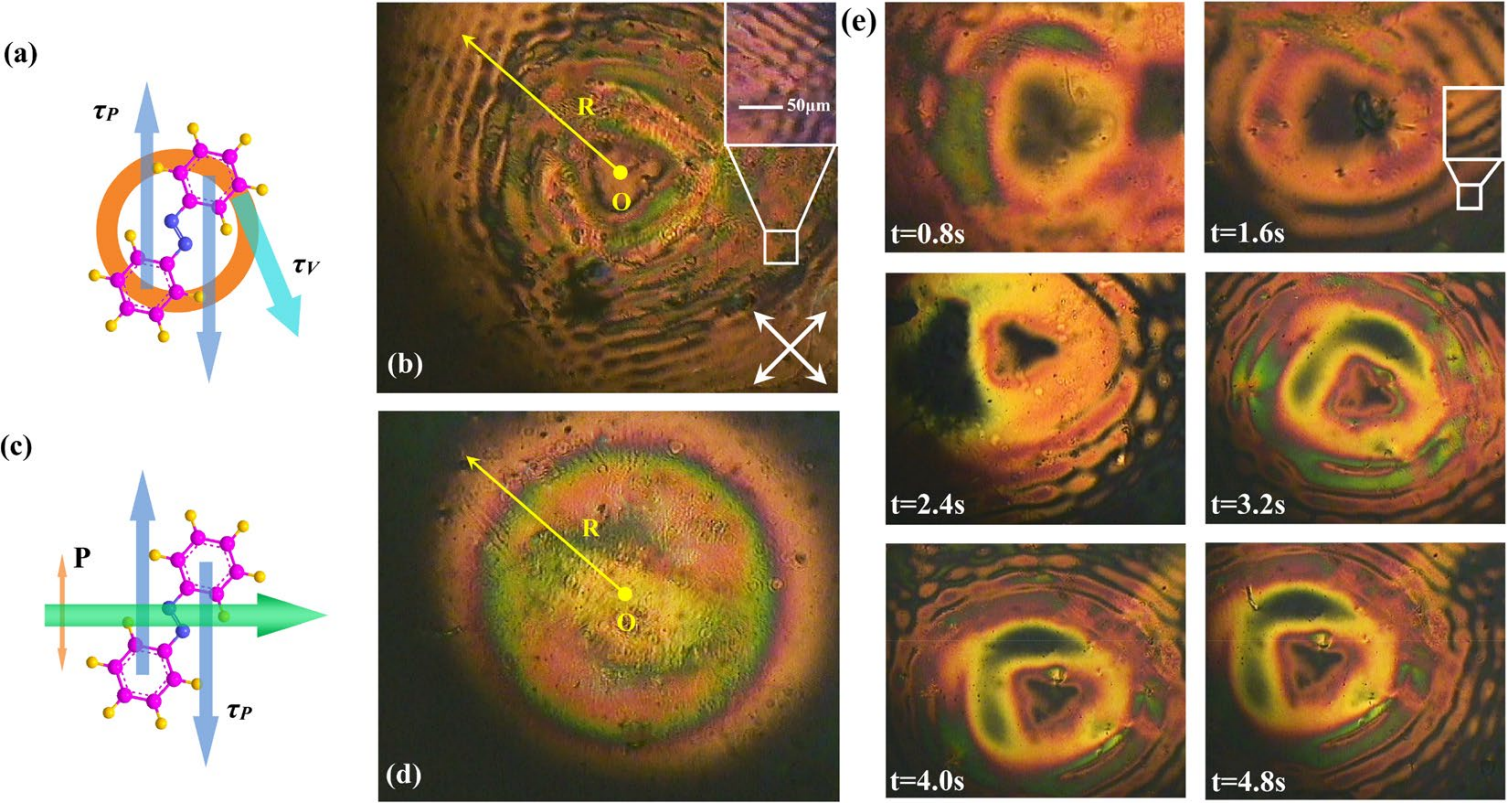
(**a**) Torques acting on the ALC induced by a phase-vortex beam. ***τ***_***V***_ and ***τ***_***P***_ are the vortex- and polarization-induced torques, respectively. (**b**) POM image of the ripple pattern within the ALC film. R is the radius of the illumination region. Transmission axis directions of the POM polarizers are presented as the crossed arrows. (**c**) Diagram of the light-matter interaction with a single Gaussian beam. P is the polarization direction. (**d**) POM image of the Gaussian excitation region without the ripple. (**e**) Sequencing POM images of the ripple formation and diffusion from 0 s to 4.8 s with an interval of 0.8 s. After the pump beam is removed, the ripple stops spreading and is stored in the ALCs.

The phase-induced polarization modulation is mainly attributed to the formation of birefringence generated by the phase vortex. Takes a kind of phase gratings, the polarization holographic grating, as an analogy^34^. As the polarization holographic grating is recorded in the material with two orthogonally circularly polarized beams, the molecular reorientation directions in the different areas rotate with the cycloidal polarization distribution of the interference light field and the periodically distributed photoinduced birefringence is formed in the film, resulting in the property of polarization modulation of the polarization holographic grating^35–37^. In this experiment, the torque generated by the phase vortex possesses the similar rotation effect^38,39^ which leads to the formation of birefringence in the ALC film, resulting in polarization modulation. In order to demonstrate this, the photoinduced torques acting on the ALCs are discussed. First, let us study the tangential torque ***τ***_***V***_ induced by the phase vortex^40^.

9 $$|{{\boldsymbol{\tau }}}_{{\boldsymbol{V}}}|=(m/\omega )\cdot Abs$$

where *ω* is the angular frequency of the pump beam, *m* is the value of TC and *Abs* is the absorption power of the ALC film. Because of the existence of ***τ***_***V***_, the vortex-induced ripple pattern in the ALC film is detected at the edge of the irradiation area by a polarizing optical microscope (POM) with crossed polarizers, as shown in Fig. 5(a,b). For the maximum transmittance, the axis directions of the POM polarizers are ±45° in respect of the s-direction, respectively. The triangle dot in the center of Fig. 5(b) corresponds to the phase singularity of *I*_2_ in Fig. 1. The POM image induced by a single Gaussian beam is also presented in Fig. 5(c,d) as a comparison, while no ripple pattern is found at the edge. Due to the tangential torque ***τ***_***V***_, ALCs are “stirred” azimuthally and the molecular reorientation direction varies periodically in the radial direction, which is illustrated by the concentric-ring-shaped brightness distribution of the POM image. Similar to the polarization holographic grating mentioned above, the photoinduced birefringence is formed through the rotation of ALCs induced by ***τ***_***V***_. The formation and diffusion process of the photoinduced birefringence is shown in Fig. 5(e). The ripple pattern starts to appear at 1.6 s with the pump light on and radially spreads outward from the center. After turning off the pump beam, the ALC arrangement is fixed and the ripple-shaped birefringence is stored in the film.

**Figure 6:**
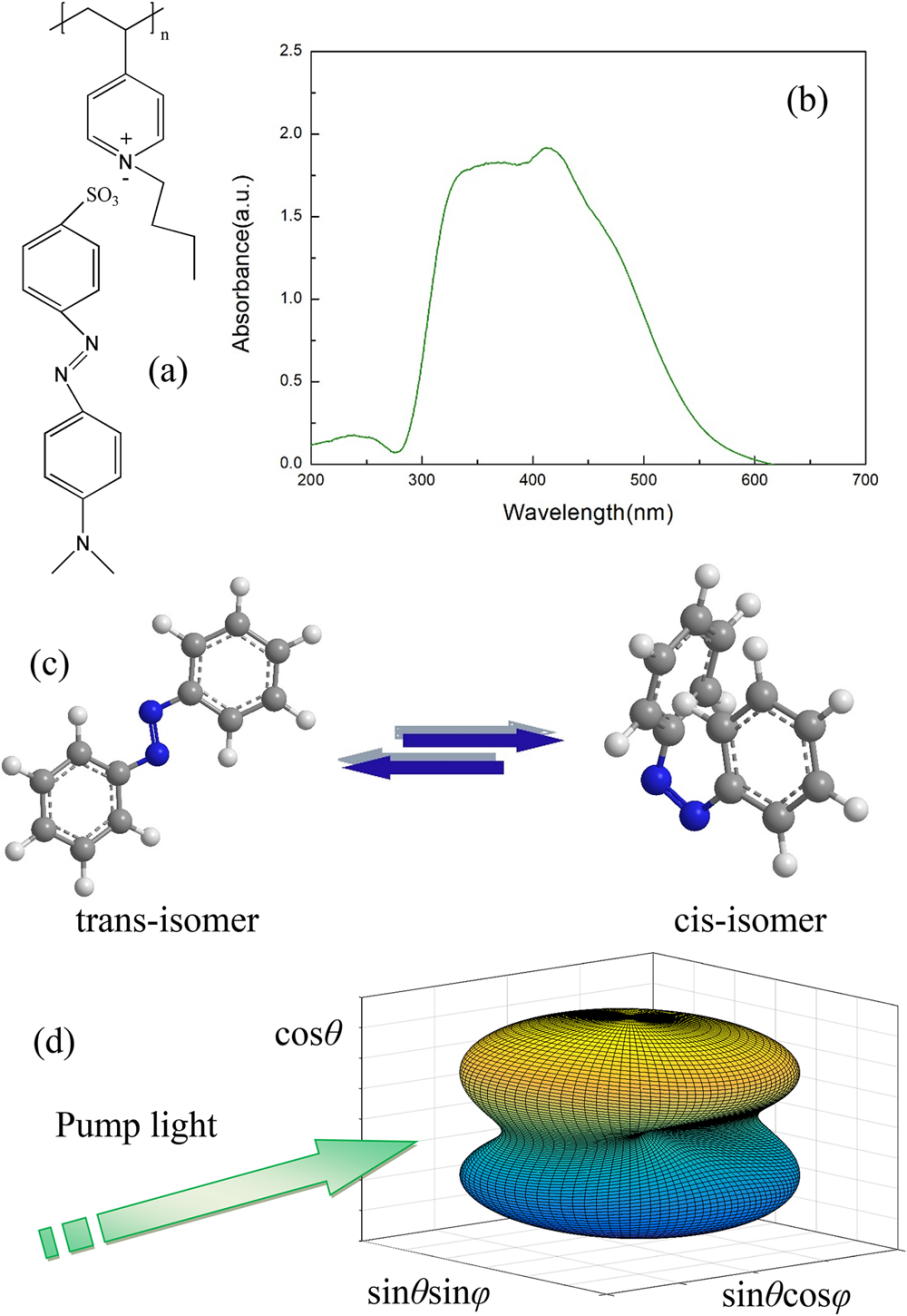
(**a**) Chemical structural formula, (**b**) absorption spectrum and (**c**) photoinduced *trans*-*cis* isomerization of the side-chain azobenzene liquid crystals. (**d**) Transition probability for an azobenzene molecule at a given orientation is presented by the radial length from the center to the surface at that orientation in the normalized spherical coordinates.

In addition to ***τ***_***V***_, the polarization-induced torque ***τ***_***P***_ acting on ALCs (see Fig. 6(a)) is also analyzed. When a polarized beam with the wavelength located in the absorption spectrum (see Fig. 6(b)) illuminates the ALC film, azobenzene groups order themselves in such a way that their orientation directions become perpendicular to the polarization direction of light through the repeated *trans*-*cis* isomerization cycles^41^, as shown in Fig. 6(c). This photoisomerization process produces intermolecular torques ***τ***_***P***_ between the azobenzene groups and liquid crystals, resulting in the reorientation of the whole molecules, which brings about the photoinduced anisotropy within the film. The polarization-sensitive absorption of the ALC can be described with the absorption cross section *σ*^42^.

10 $${\sigma }={{\rm{\sigma }}}_{{1}}\cdot ({a}^{2}{\rm{c}}{\rm{o}}{{\rm{s}}}^{2}\theta +{b}^{2}{\rm{s}}{\rm{i}}{{\rm{n}}}^{2}\theta \,{\rm{c}}{\rm{o}}{{\rm{s}}}^{2}\varphi )+{{\rm{\sigma }}}_{{2}}\cdot [{a}^{2}{\rm{s}}{\rm{i}}{{\rm{n}}}^{2}\theta +{b}^{2}(1-{\rm{s}}{\rm{i}}{{\rm{n}}}^{2}\theta \,{\rm{c}}{\rm{o}}{{\rm{s}}}^{2}\theta \,{\rm{c}}{\rm{o}}{{\rm{s}}}^{2}\varphi )]$$

where *σ*_1_ and *σ*_2_ are the parallel and vertical absorption cross sections respectively, *a* and *b* are the normalized major and minor semi-axes of the polarization ellipse and (*θ*, *φ*) are spherical coordinates. The dependence of the absorption cross section on the polarization of pump light is demonstrated in Fig. 6(d). Considering the nonlinear response of the film caused by the electric field ***E*** of light, ALCs are forced by the polarization-induced torque ***τ***_***P***_^43^

11 $${{\boldsymbol{\tau }}}_{{\boldsymbol{P}}}=\frac{\Delta \mu }{4\pi }({\boldsymbol{n}}\cdot {\boldsymbol{E}})[{\boldsymbol{n}}\times {\boldsymbol{E}}]$$

where ***n*** is the orientation direction of the ALC and Δ*μ* = *μ* − *μ*_*eff*_ with *μ* and *μ*_*eff*_ being the dielectric and effective optical anisotropy, respectively. Within the Gaussian-Gaussian interference area, the ALC reorientation direction is perpendicular to the polarization direction where the light intensity is strong because of ***τ***_***P***_, while the ALC arrangement is disordered in the region with weak light intensity, leading to the periodic refractive-index variation in the ALC film.

Therefore, for the holographic grating generated by Gaussian-Gaussian interference without the extra phase, only ***τ***_***P***_ exists and the resultant interference light shows constant polarization and modulated intensity in space. Due to the connection between the sinusoidally distributed light intensity and the degree of molecular order, the refractive index of the material in different regions varies periodically and an amplitude grating is generated^44^. The amplitude grating is not able to manipulate the polarization state of the incident light. However, in terms of VBG, ALCs are controlled by the total optical torque ***τ***_***opt***_ = ***τ***_***P***_ + ***τ***_***V***_ and the rearrangement current is generated within the excitation area^45^. Because the competition between ***τ***_***P***_ and ***τ***_***V***_ depends on the fork-shaped intensity distribution of the vortex-Gaussian interference field, the ALC orientation in center area is supposed to be controlled by ***τ***_***P***_. As the light intensity is attenuated off the center, ***τ***_***V***_ starts to exert an influence on ALC arrangement at the edge, leading to the formation of birefringence. As a result, a double-layer structure is formed within the vortex-Gaussian interference field because a phase-vortex-induced birefringence is added to the refractive-index grating, which makes the VBG possess the function of polarization modulation.

### Double-layer structure of VBG

**Figure 7:**
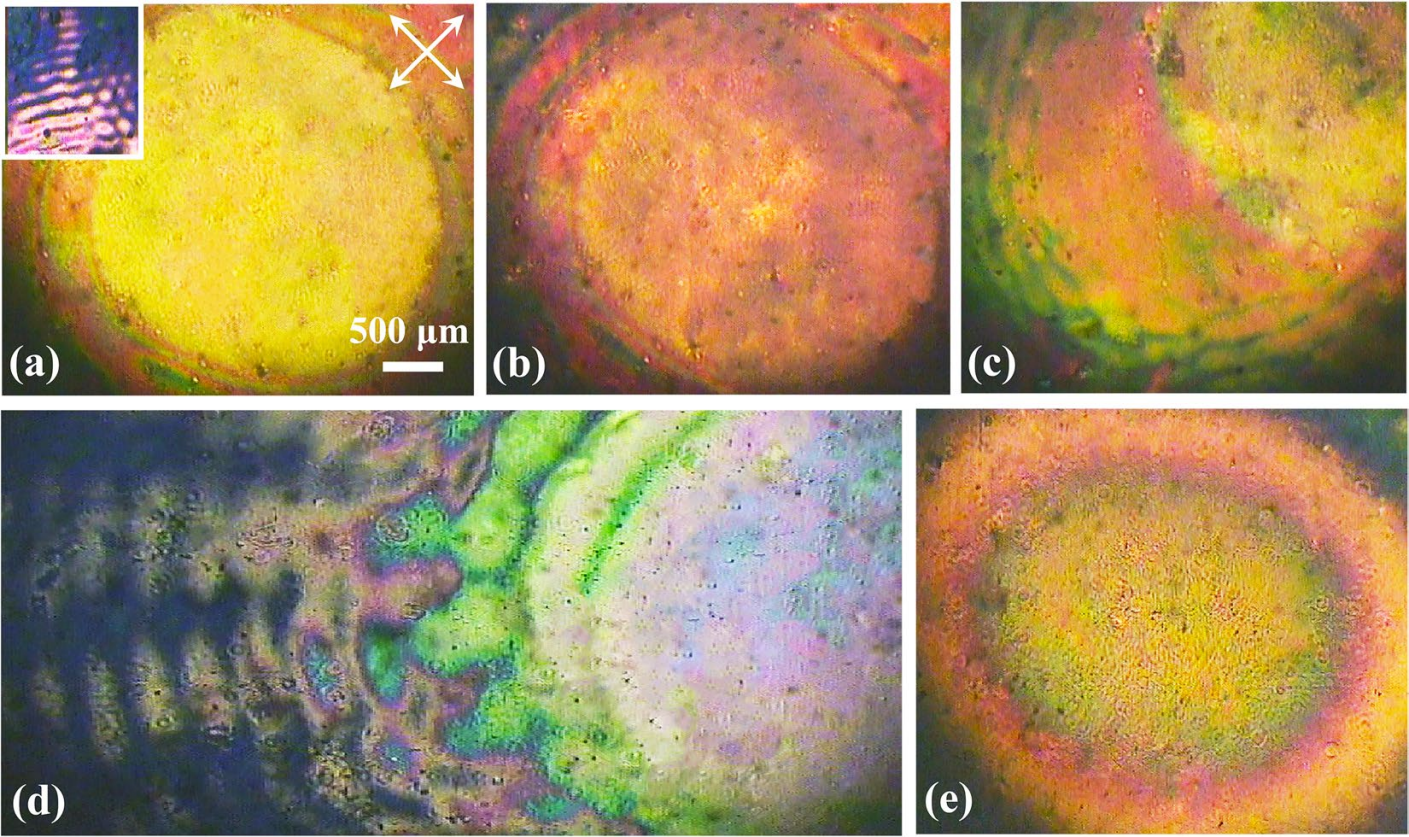
POM images of the VBG consisting of the polarization-controlled center and vortex-induced ripple edge. The intensity ratio between *I*_1_ and *I*_2_ is (**a**) 1:1, (**b**) 1:2, (**c**) 1:3 and (**d**) 1:4. Inset: enlarged image of the ripple pattern and the transmission axis directions of the POM crossed polarizers. (**e**) POM image of the Gaussian-Gaussian hologram is detected as a comparison.

To verify the discussion above, various VBGs are recorded through the holographic interference of a Gaussian beam and a vortex beam with different intensity ratios, and the ALC patterns are detected by the POM, as shown in Fig. 7. The axis directions of the POM polarizers are still ±45° for the maximum transmittance. The recorded double-layer hologram consists of two parts, the polarization-controlled center area and the vortex-controlled ripple edge (see the inset of Fig. 7(a)). In terms of the vortex-Gaussian interference, the phase vortex cannot be totally neutralized and the ALCs are still able to be forced by ***τ***_***V***_^46^. With the intensity of *I*_2_ in Fig. 1 increasing from 1:1 to 1:4 (*I*_1_:*I*_2_), the effect of ***τ***_***V***_ is enhanced and the ripple edge is widened from Fig. 7(a,d). Moreover, the molecular reorientation in the center keeps being controlled by ***τ***_***P***_ and the area is always free of ripples, regardless of the intensity ratio variation. For the Gaussian-Gaussian interference in Fig. 7(e), the ALC arrangement is only forced by ***τ***_***P***_ and the outer ripple layer is not formed. The double-layer structure of VBG detected by the POM is consistent with the pattern analysed by the competition between ***τ***_***V***_ and ***τ***_***P***_.

**Figure 8:**
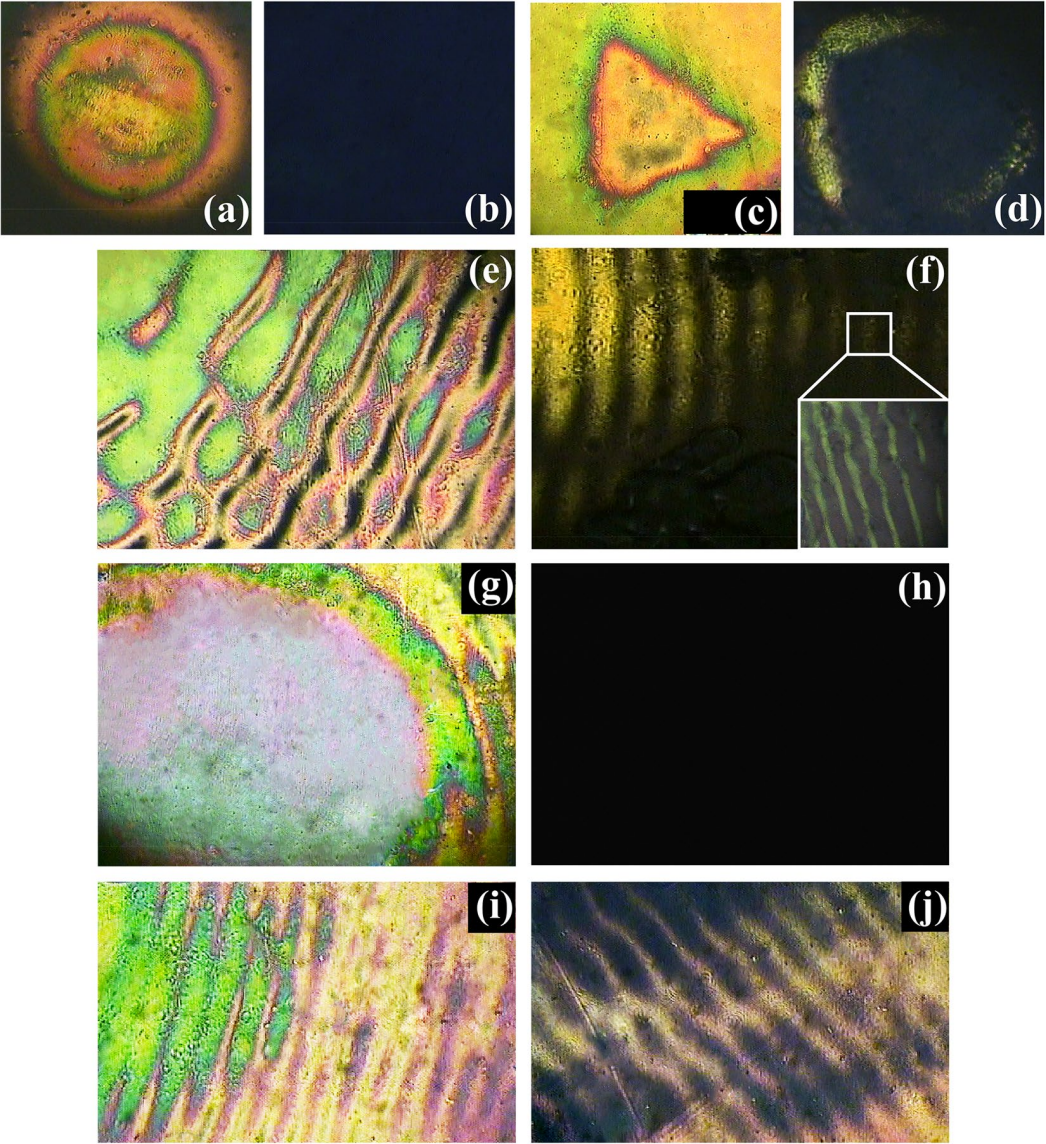
POM images of the ALC patterns induced by (**a**) a single Gaussian beam, (**c**) the center and (**e**) the edge of a single vortex beam, (**g**) the center and (**i**) the edge of the vortex-Gaussian interference field under the condition of the maximum transmittance through the crossed polarizers. After the ALC film is rotated 45° clockwise, POM images of (**a**,**c**,**e**,**g**,**i**) change to (**b**,**d**,**f**,**h**,**j**), respectively.

In order to distinguish the effects between the vortex-induced birefringence modulation and polarization-induced alignment direction modulation on the ALC film, contrast POM images are detected when the film is rotated 45° clockwise in respect of the direction where the transmittance of the crossed POM polarizers is maximum. Theoretically, when the ALC orientation direction is parallel to one of the crossed polarizers (perpendicular to another), the transmittance of the crossed polarizers is 0. In terms of the alignment direction modulation induced by ***τ***_***P***_, the ALCs are arranged in a single direction and nothing can be observed when the film is rotated 45°. In contrast, the birefringence modulation is mainly induced by the tangential force ***τ***_***V***_. The molecular orientation affected by ***τ***_***V***_ is not uniform so that the ripple pattern is still supposed to be observed after the film rotation. To demonstrate this, the POM images before and after the film rotation are presented in Fig. 8. Under the condition of the illumination of a single Gaussian beam in Fig. 8(a,b), the brightness of the POM pattern drops dramatically with the film being rotated 45°, which agrees with the prediction. From Fig. 8(c–j), it can be seen that the POM patterns at the center of the vortex light and VBG also disappear as the film is rotated 45°. Though the brightness of the POM images at the edge decreases as well, the vortex-induced ripple patterns are still able to be detected. The experimental results are consistent with the discussion that the ALC arrangement in the VBG center is controlled by ***τ***_***P***_ and the ripple-shaped birefringence is generated by ***τ***_***V***_. Therefore, different from the periodic distribution of the refractive index formed by the interference of two Gaussian beams with the same polarization state, the vortex-induced birefringence (ripple pattern) is added to the amplitude grating (polarization-controlled center), which contributes to the property of polarization modulation of VBG. It should be noted that polarization modulation can also be achieved when TC takes other integers. Furthermore, the polarization modulation depth of VBG is affected by many factors, such as the value of TC of the vortex recording beam, intensity ratio between the Gaussian beam and the vortex beam, polarization states of the recording beams and so on. For example, when we change the value of TC, the polarization state of the diffraction light from VBG is different from the condition of TC = 3. The reason is that ***τ***_***V***_ becomes larger as the value of TC increases^40^, leading to the change of the vortex-induced birefringence in the ALC film.

## Conclusions

In general, we have successfully realized all-optical polarization modulation through adding an extra phase to the recording light with a spiral phase plate. The VBG is recorded by means of the holographic interference of a Gaussian beam and a phase-vortex beam, resulting in the formation of the periodically distributed photoinduced anisotropy in the ALC film. According to the POM images of VBG, a double-layer structure is generated, including the polarization-controlled center and the vortex-induced ripple edge. On the contrary, no ripple pattern is detected under the condition of Gaussian-Gaussian interference. Therefore, in addition to the periodically distributed refractive index induced by the polarization-induced torque ***τ***_***P***_ in the ALC film, phase-vortex-induced birefringence is also generated at the edge of the recording area, which is demonstrated by the POM ripple patterns in the outer layer. The formation of ripple patterns is attributed to the molecular rotation which is analyzed through the competition between ***τ***_***P***_ and ***τ***_***V***_. Due to the generation of photoinduced birefringence, the polarization state of the incident light is able to be modulated by the recorded VBG and the property of polarization modulation is calculated through Jones matrices. Moreover, the experimental results could enrich the connotation between optical parameters and offer an alternate way of all-optical polarization modulation.

## Methods

### Material preparation

The sample is a kind of supermolecular materials synthesized through the ionic self-assembly of poly ionic liquid and azobenzene dyes. The preparation process has been reported in ref. ^47^. For the preparation of ionic self-assembly complex, 2 mg**/**ml poly ionic liquid aqueous solution is added to methyl orange aqueous solution at the molar charge ratio of 1:1. The precipitated complex is filtrated and washed several times with doubly distilled water, then dried in vacuum at 60 °C for 12 h. The complex powder melts at 180 °C and the Schlieren textures appear during cooling.

## Data Availability

The datasets generated during and/or analysed during the current study are available from the corresponding author on reasonable request.
